# PVDF Sensor Stimulated by Infrared Radiation for Temperature Monitoring in Microfluidic Devices

**DOI:** 10.3390/s17040850

**Published:** 2017-04-13

**Authors:** Salvatore A. Pullano, Ifana Mahbub, Syed K. Islam, Antonino S. Fiorillo

**Affiliations:** 1 Department of Health Sciences, University Magna Græcia of Catanzaro, Viale Europa, 88100 Catanzaro, Italy; nino@unicz.it; 2 Department of Electrical Engineering and Computer Science, University of Tennessee, 1520 Middle Drive, Knoxville, TN 37996, USA; imahbub@vols.utk.edu (I.M.); sislam@utk.edu (S.K.I.)

**Keywords:** pyroelectric devices, temperature sensor, system-on-a-chip, ferroelectric materials, thin film sensors

## Abstract

This paper presents a ferroelectric polymer-based temperature sensor designed for microfluidic devices. The integration of the sensor into a system-on-a-chip platform facilitates quick monitoring of localized temperature of a biological fluid, avoiding errors in the evaluation of thermal evolution of the fluid during analysis. The contact temperature sensor is fabricated by combining a thin pyroelectric film together with an infrared source, which stimulates the active element located on the top of the microfluidic channel. An experimental setup was assembled to validate the analytical model and to characterize the response rate of the device. The evaluation procedure and the operating range of the temperature also make this device suitable for applications where the localized temperature monitoring of biological samples is necessary. Additionally, ease of integration with standard microfluidic devices makes the proposed sensor an attractive option for in situ analysis of biological fluids.

## 1. Introduction

One of the most important aims of the development of a biomedical system-on-a-chip (SoC) is to achieve successful integration of sensors and actuators into a low-cost device which will provide a wide range of functionalities required for most applications [1]. In recent literature, several devices implementing on SoC platform have been reported for detection of genetic variation, integrated polymerase chain reaction, and electrophoretic analysis [2,3,4]. These devices usually have low thermal inertia—a characteristic that allows for rapid changes in the temperature of the biological samples. This phenomenon occurs frequently and requires innovative technological solutions for its rapid, localized, and accurate assessment. It is particularly relevant in the case of fast temperature changes (in the millisecond time scale) where it is known to be one of the primary causes of errors [5,6,7,8]. Various transducer technologies have been employed for in situ temperature measurement such as thermocouples, thermistors, and thermochromic liquid crystals, but there is still an increasing demand for low-cost contact sensors in micro-scale applications [9,10,11,12,13]. The performance of a SoC device can be improved by incorporating integrated temperature transducer with dimensions of few µm^2^ of area and featuring fast response, high accuracy, easy integration, low power consumption, and improved reliability. Among various available transducer technologies, the polymer sensor is one of the fastest growing technologies for commercialization and has maturated for practical applications from a long list of candidates. Ferroelectric polymers such as polyvinylidene fluoride (PVDF) is a polarized fluoropolymer that, in its polar form (β-phase), demonstrates strong and stable piezoelectric and pyroelectric activities. Thermal transducers employing a thin pyroelectric film of PVDF can be designed to provide fast response time. In addition, the pyroelectric voltage coefficient of PVDF is about an order of magnitude larger than that of other pyroelectric materials such as lead zirconate titanate (PZT) or barium titanate (BaTiO_3_) [14].

The pyroelectric transducer provides an electrical response due to the change in its temperature and therefore typically requires an adjunctive reference sensor to provide information about the target temperature. However, recent literature reported efforts on the development of high performances pyroelectric temperature sensors that avoid supplementary components representing a promising technology, particularly for medical applications [15,16,17]. In previous work, PVDF-based pyroelectric sensors were investigated for monitoring of fast temperature variations in microfluidic devices. Due to the dynamic nature of the PVDF transducer, the device only provided information on the temperature gradient but not the initial and final temperature values [18,19,20]. Moreover, the respectable degree of compatibility of PVDF with most of the polymeric substrates used for fabrication of disposable devices (e.g., polymethylmethacrylate, etc.) makes it suitable for integrated sensor applications [21,22].

In this paper, a modified pyroelectric transducer is investigated for overcoming previous limitations in the evaluation of the absolute temperature inside a microfluidic channel. Moreover, a thermal model based on the analysis of heat transfer mechanism is developed for the characterization of the transducer. The pyroelectric contact thermometer is composed of a thin film of PVDF coupled with a microfluidic device and includes an infrared (IR) light source and a charge amplifier that generates a suitable voltage signal in response to the electrical charge [20,23,24,25]. As a consequence of the modified design of the transducer, the use of mechanical components (such as shutters) and adjunctive reference sensor (e.g., thermocouple) are eliminated in the proposed configuration. Simulations of the developed thermal model were compared with the experimental results for the validation of the proposed sensor. The working bandwidth of the system (0.8 to 49.7 Hz) can be adjusted for specific applications by modifying the geometry of the transducer and the front-end circuitry. Comparison of the proposed sensor with other temperature sensors reported in literature shows a faster response time along with a smaller footprint thereby making the proposed sensor suitable for SoC applications.

## 2. Pyroelectric Sensor Design

### 2.1. Device Fabrication

**Figure 1:**
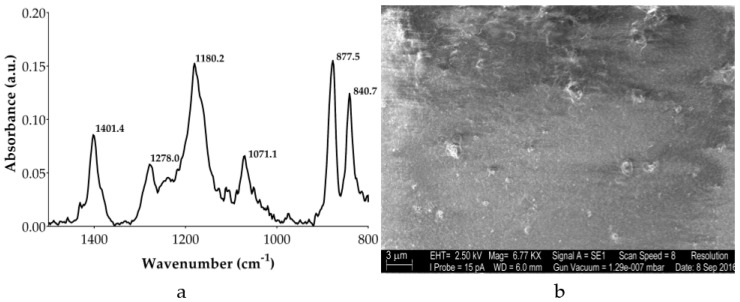
Polyvinylidene fluoride (PVDF) characterization obtained by (**a**) Fourier transform infrared spectroscopy and (**b**) scanning electron microscopy.

The device was fabricated using a uniaxially stretched 28 µm thick sheet of PVDF (Measurement Specialties, Hampton, VA, USA) which is a ferroelectric polymer synthesized in polycrystalline form and exhibits a strong and stable pyroelectric effect with the direction of the polarization axis being perpendicular to both surfaces. Attenuated total reflection Fourier transform infrared (ATR-FTIR) spectroscopy has been used to characterize molecular conformation of PVDF. Among different crystalline structures of PVDF, β-phase is the most interesting for technological purpose due to its stronger piezoelectric and pyroelectric properties [26]. In particular, higher β-phase conformation film provides higher yield within the thin film of PVDF. Figure 1a shows a representative FTIR-ATR spectrum of PVDF film in which peaks around 1278 and 1238 cm^−1^ highlight the film containing both β-phase and γ-phase while higher peaks at about 840 cm^−1^ indicate a PVDF film with a higher content of β-phase crystalline structures. Moreover, analysis performed along the sample showed a very good degree of uniformity, which thus reduced differences in pyroelectric response all along the film. SEM analysis of Figure 1b underlines a good surface quality in terms of roughness and density, which leads to a good quality PVDF film for thermo-electrical application [27].

**Figure 2:**
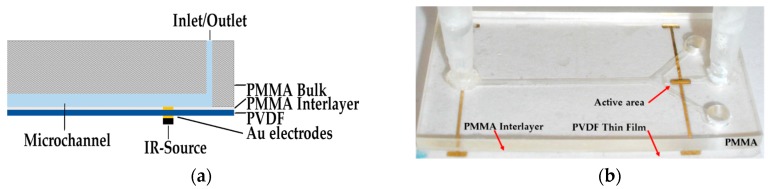
(**a**) Cross-section and (**b**) picture of the microfluidic device containing the micro-milled PMMA bulk and the PVDF thin film on which the gold metallizations are deposited to serve as active element and electrical connections to the read-out electronic circuit.

Sputtered gold (Emitech K650X, EM Technologies Ltd., Ashford, UK) was used to obtain 300 nm-thick electrodes on both faces of the sheet at a temperature sufficiently lower than the transition temperature to avoid depolarization. Gold metallization layers were fabricated to be overlapped only in the area over the microchannel (i.e., active transducer area), which in the actual arrangement was measured to be 2 × 0.5 mm^2^. The metallization outside the microchannel was not overlapped and just provided electrical pathways for signal acquisition and analysis. A thin black layer of graphite was sputtered onto the active area and an infrared LED source was then fixed in contact with the transducer. Each layer added onto PVDF sheet creates an additional heat path that is undesirable since it reduces the efficiency of delivering heat to/from the pyroelectric element limiting the sensitivity and the resolution. In order to reduce the additional heat paths, the gold metallization and the graphite layer (about 500 nm in thickness) were designed to be thinner compared to the PVDF film. Moreover, the graphite layer allows most of the infrared energy transmitted by the IR source to be absorbed and not reflected by the metallic layer. The transducer was permanently fixed on the surface of the microfluidic chip through a PMMA interlayer that electrically insulated it, while the external contact was realized via ultrasound soldered gold conductors. The interlayer was realized with a solution of PMMA (Röhm, Milano, Italy) and Anisole 99% (Carlo Erba, Milano, Italy) that was first spin-coated (1 µm in thickness) onto the lower PVDF surface as shown in Figure 2 [19]. Anisole was used as a solvent for PMMA with reduced health-related issues.

The PMMA bulk hosts the holes for inlet and outlet connections. PMMA and PVDF layers were sealed by means of a liquid PMMA interlayer spun onto the PVDF and then pressed toward the PMMA bulk.

### 2.2. Pyroelectric Charge Generation

The pyroelectric effect is the manifestation of the temperature dependence of the spontaneous polarization in certain anisotropic solids. When the temperature *θ* of a pyroelectric material is changed, a voltage is produced across the sensor [28]. Assuming the sample heating rate to be significantly smaller than the thermal diffusivity time, the sample can be considered to be uniformly heated and thus the generated voltage *dV_p_* or equivalently the charge *dQ_p_*, is described by,

(1) $$dV_{p}=p_{V}hd\theta ,\;dQ_{p}=p_{Q}Ad\theta$$

where *p_V_* and *p_Q_* are the voltage- and the charge-pyroelectric coefficients and *h* and A are the thickness and the area of the material, respectively. Pyroelectric coefficients are related to each other by the ratio *p_Q_/p_V_* which corresponds to the permittivity of the material, *ε*. The response is achieved when the surface of the material is perpendicular to the direction of polarization [29]. A pyroelectric response is stimulated on a pyroelectric thin film, metallized on both surfaces, with an IR impulse to impose a temperature difference between the top absorbing layer and the lower face, *θ_P_*(*t*) *= θ_U_*(*t*) *− θ_L_* (taken as reference during IR stimulation) [30]. If the thermal wave that propagates through the outer absorbing metallic layer is not taken into account then considering the thermal diffusivity of the metal (e.g., gold) to be much higher than that of the pyroelectric material and at the same time its thickness to be very small, *θ_P_*(*t*) following an infrared impulse is dictated by,

(2) $$C_{th}\frac{d\theta_{p}(t)}{dt}+\frac{1}{R_{th}}\theta_{p}(t)=\alpha P(t)$$

where *C_th_* and *R_th_* are the thermal capacitance and resistance of the PVDF film, respectively, *α* is the absorption coefficient of the outer metallic layer on which the radiation is incident and *P*(*t*) is the radiation power [30,31]. The response time of the pyroelectric transducer is related to its thermal time constant, *τ_th_* = *C_th_*·*R_th_*. By using Laplace transform, the convolution theorem, and inverse Laplace transform, we can rewrite Equation (2) as,

(3) $$\theta_{p}(t)=\frac{\alpha \tau_{th}U}{C_{th}}\left(e^{\frac{T_{IR}}{\tau_{th}}}-1\right)e^{\frac{-t}{\tau_{th}}}$$

where the term *U* represents the energy of the infrared source. Equation (3) highlights that *θ_P_*(*t*) rises almost instantly to its maximum value following the IR stimulation (*θ_P_*(0)). Afterward, it decreases within a time defined by the thermal time constant, *τ_th_*. By using first order Taylor series expansion and considering the step duration of the IR source, *T_IR_* << *τ_th_*, Equation (3) becomes,

(4) $$\theta_{p}(t)=\frac{\alpha \tau_{th}U}{C_{th}}\left(e^{\frac{T_{IR}}{\tau_{th}}}-1\right)\left(1-\frac{t}{\tau_{th}}\right)$$

**Figure 3:**
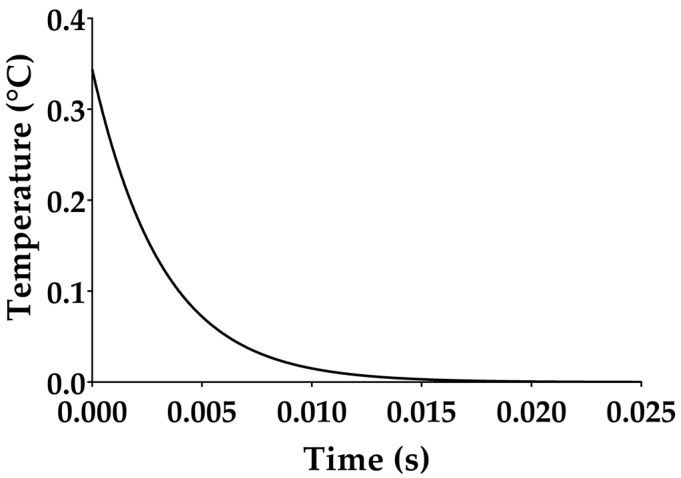
Analytical temperature difference *θ_p_*(*t*) between the top absorbing layer, *T_U_*(*t*), and the lower face, *T_L_*, following a step-shaped infrared (IR) radiation source of 50 mW with a time duration *T_IR_* = 0.3 ms and considering the parameters reported in Table 1. By considering similar radiation impulse, according to Equation (4), the higher the thermal time constant, the longer the return to thermal equilibrium (*θ_U_*(*t*) *= θ_L_*).

Temperature evolution, *θ_P_*(*t*), following a given IR laser impulse, is reported in Figure 3 using the same geometrical dimensions (i.e., length width and thickness) and thermal properties (i.e., specific heat, thermal conductivity) of the fabricated device and following Equation (4). *C_th_* and *R_th_* mainly affect the return to thermal equilibrium of the pyroelectric film. In particular, higher the value of *τ_th_* the slower is the return to the thermal equilibrium. 

Stimulating the pyroelectric transducer with an IR source impulse with *T_IR_* << *τ_th_*, a current is thus generated, which decreases exponentially following the thermal time constant of the transducer. Considerations can be made regarding the generation of a pyroelectric charge using Equation (4) under the condition *T_IR_* << *τ_th_* and neglecting the quadratic terms, which according to Equation (1) becomes:

(5) $$Q_{p}(t)≅\frac{pA\alpha U}{C_{th}}t$$

Therefore, based on the above-mentioned assumption, the charge generated is a linear function of time.

### 2.3. Charge Amplifier

**Figure 4:**
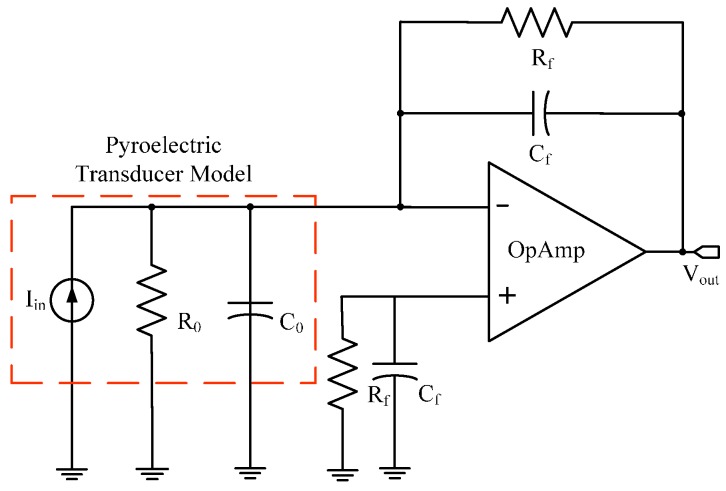
Schematic diagram of the designed charge amplifier.

A charge amplifier implemented as a current integrator integrates a pulse of current and produces an output voltage *V_o_* proportional to the pyroelectric charge through the factor 1/*C_f_*. Feedback capacitance is designed to make the transfer process independent of the transducer and any other stray capacitances, while feedback resistance provides a current path for the feedback capacitor to be reset [32,33]. It is characterized by a high input and low output impedance. The input impedance of the charge amplifier is composed of the input impedance of the operational amplifier as well as the inherent PVDF capacitance *C*_0_ and a leakage resistance *R*_0_. The gain of the charge amplifier is also influenced by the charge transfer from *C*_0_ to *C_f_* [34,35]. Figure 4 shows the schematic diagram of the charge amplifier with a feedback capacitance (*C_f_* = 1 nF) and resistance (*R_f_* = 100 MΩ) which is used to convert the temperature-dependent charge collected on the metallized surfaces into a proportional voltage.

OPA124 op-amp is used for implementation of the front-end charge amplifier, which provides a high gain-bandwidth-product (GBW = 1.5 MHz), low bias current (*I_b_* = 1 pA max.), high slew-rate (SR = 1.6 V/μs), and high DC open loop gain (*A_vol_* = 125 dB). Moreover, its high input impedance (*Z_in_* = 10^13^ Ω ||1 pF) avoids bleed-off of the charge on the feedback capacitor and the low bias current prevents the feedback capacitor from charging and discharging at excessive rates [19]. *C*_0_ and *R*_0_ have been evaluated at low frequencies using a Keithley 4200-SCS source meter resulting in *C*_0_ = 5.01 ± 0.09 pF and *R*_0_ = 213.8 ± 53.8 GΩ at 1.32 Hz. The electrical time constant *τ_e_* = *R_in_·C_in_* depends on the equivalent input resistance *R_in_* = *R_f_*/(1 + *β A*)||*R*_0_ shunted by the input equivalent capacitance *C_in_ =* *C_f_* (1 + *β A*)||*C*_0_, where *β =* −1/*Z_f_* is the feedback factor and *A =* −*A_vol_Z_f_* is the open loop gain (i.e., *τ_e_* = 100 ms) [20]. *C_f_* and *R_f_* on the non-inverting input are used to balance the source impedances (both resistive and reactive) in order to minimize input bias current errors and output noise together with shielding through input guard [34]. Both the thermal and the electrical time constants define a frequency band of the transducer (in the actual configuration is 0.8–49.7 Hz). By neglecting the output resistance and assuming an ideally infinite input resistance of the operational amplifier, Equation (6) can be written as,

(6) $$V_{o}=-\frac{Q_{p}}{C_{f}}e^{-t/\tau_{e}}$$

Here, the signal amplitude decreases following the time constant, *τ_e_* and the maximum value of the response is a measure of the temperature variation.

### 2.4. Heat Transfer Process

**Figure 5:**
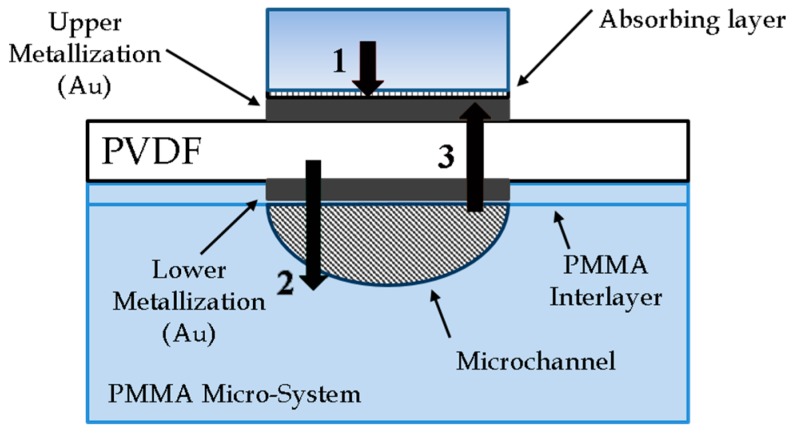
Cross section of the device highlighting with the black arrows the heat transfer mechanisms involving (**1**) infrared thermal absorption, (**2**) thermal radiation, and (**3**) thermal conduction.

Recently, there has been a great deal of interest in the use of pyroelectric effect for making thermal measurements and detecting electromagnetic radiations [36,37,38]. Low-temperature differential measurements, such as the evaluation of specific heat require high sensitivity and minimum joule heating, are readily provided by the pyroelectric devices [39,40]. There are different ways to characterize a thermometer, especially in the initial evaluation phase, by developing a correlation between the physical properties of the transducer with variations in temperature or by the evaluation of a thermal property of the material itself [41]. From the thermal point of view (see Figure 5), a pyroelectric transducer mounted on a microfluidic chip is governed by the following mechanisms: IR absorption, thermal radiation and thermal conduction. The absorption of the IR radiation on the outer layer generates heat that results in the rise of the temperature and a net heat flow is described as,

(7) $$\Delta W_{IR}=A\alpha I\Delta t$$

where A is the surface, *α* is the surface absorption coefficient and *I* is the irradiance. The heat transfer mechanisms of PVDF transducer involving thermal radiation and the thermal conduction are described using the following equations, respectively:

(8) $$\Delta W_{TR}=A\delta \sigma \theta^{4}\Delta t$$

(9) $$\Delta W_{Cond}=\frac{1}{R_{th}}\theta_{p}(t)\Delta t$$

where *δ* is the surface emissivity and *σ* is the Stefan-Boltzmann constant. The overall thermal effect, which gives rise to temperature variations, is described as follows:

(10) $$\Delta W_{tot}=A\alpha I\Delta t-A\delta \sigma \theta^{4}\Delta t-\frac{1}{R_{th}}\theta_{p}(t)\Delta t=C_{th}\theta_{p}(t)$$

Therefore, the response to an infrared pulse is dependent on the absolute temperature according to Equation (10). The IR absorption term is expected to be constant due to the intensity of the IR source and the thermal radiation term depends on *θ*^4^. The term that takes into account the thermal conduction is expected to be constant and small if compared with the previous terms, as the total radiant flux is maintained at a low value (very small temperature change on the pyroelectric element due to IR source if compared with the target temperature). Therefore, increasing the target temperature of the device results in a heat flow dominated by thermal radiation mechanism (i.e., *θ*^4^). By taking into account the contribution of the charge amplifier, Equation (10) can be rewritten as,

(11) $$\left(-\frac{V_{o}}{pA}C_{f}\right)C_{th}=A\alpha I\Delta t-A\delta \sigma \theta^{4}\Delta t-\frac{1}{R_{th}}\theta_{p}(t)\Delta t=C_{th}\theta_{p}(t)$$

**Figure 6:**
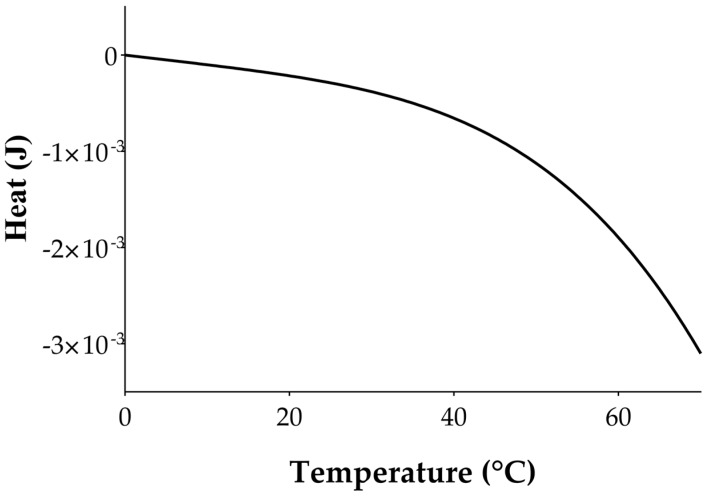
Temperature dependence of the response of the pyroelectric sensor to the heat transfer mechanism. For a given time of evaluation, *t*_0_ provides the relationship between the output voltage *V*(*t*_0_) and the temperature of the microchannel.

For a given Δ*t*, Equation (11) relates the output voltage of the pyroelectric sensor to its temperature. Figure 6 shows the analytical temperature dependence of *V_o_* by plotting Equation (11) using the parameters reported in Table 1 and by varying the temperature from 0 °C to 65 °C. 

Therefore, considering Equation (11), the microchannel temperature can be evaluated exploiting the characteristic of the pyroelectric element that is faster compared to the traditional integrated sensors because there is no latency time for the thermalization. In fact, pyroelectric response takes place instantaneously if thermal equilibrium is perturbed (inducing a difference in temperature *θ_p_*(*t*)), while other temperature sensors (e.g., thermocouples, thermistors) need the establishment of thermal equilibrium prior to the evaluation of the temperature. Concerning the dimension of the transducer, a large-sized transducer leads to larger thermal capacitance caused by the volume of the larger transducer. Consequently, the transducer is characterized by higher thermal inertia and a larger amount of heat will be required to bring the transducer to the target temperature. Thus, the lower the value of the thermal capacitance, the less significant the change in temperature of the target object.

## 3. Discussion

**Figure 7:**
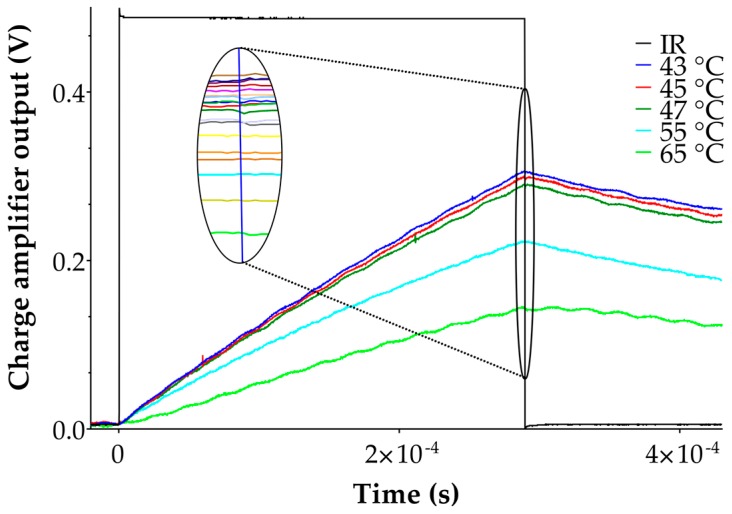
Experimental data obtained at five different target temperatures using the heating chamber. The square signal in black represents the signal generated for the IR-LED stimulation. The colored signals represent the output of the sensor at different target temperature. The slope in the time interval [*0*, *T_IR_*], varies depending on the microchannel temperature (at higher temperatures the response slope decrease). All the responses obtained in the range 25–65 °C are highlighted and the maximum points of the responses at *t* = *T_IR_* decrease according to Equation (11).

Two experimental setups were developed in order to carry out the measurements required for the necessary validation of the analytical model. The first was the adjustment of the sensor in an environmental test chamber maintained at a constant temperature (Delta Design, Delta 9023). In this case, the obtainable temperature accuracy is ±1.0 °C whilst the temperature deviation after stabilization is ±0.1 °C. The second setup consisted of a hot plate (Torrey Pines Scientific^®^, HS40, Carlsbad, CA, USA) that is thermally controlled by a Platinum RTD and is characterized by an accuracy of ±1%. Similar results were obtained by both setups, although the environmental test chamber yielded results that were less prone to electromagnetic noise (working as Faraday cage–like) and were more reliable for maintaining the channel temperature at the required level. The pyroelectric response was acquired by the front-end charge amplifier and then recorded by an Agilent MSO X3054A oscilloscope. A light-emitting diode operating at 850 nm wavelength was used as the infrared source, while a signal generator was used for the infrared impulse modulation (Agilent 33220A, Santa Clara, CA, USA) and was set at a suitable frequency, duty cycle, and amplitude. The IR diode was a high-power LED with an emission angle of ±3° and a maximum radiant flux of 50 mW which irradiates an area of 10^−6^ m^2^. The instruments were warmed up for 2 h prior to the experiment in order to prevent any temperature variation that might cause a drift in the measurement results. Experimental data obtained for the temperature range of 25–65 °C highlights the fact that the behavior of the sensor at each temperature is governed by both the thermal (rise phase) and the electrical (fall phase) characteristics of the sensor response. Figure 7 shows pyroelectric responses recorded at the charge amplifier output choosing *T_IR_ =* 0.3 ms (with *T_IR_ < τ_th_*). The results indicate that *dV_o_*/*dt* in the time frame considered decreases with the increase in the temperature.

**Figure 8:**
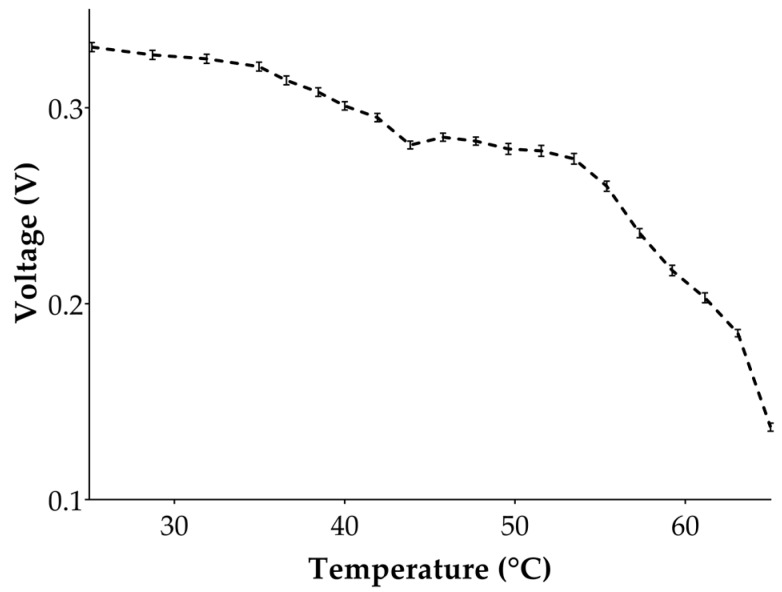
Calibration curve of the pyroelectric sensor performed inside the microfluidic channel in the temperature range 25–65 °C (as reported in the insert of Figure 7). Charge amplifier voltage are evaluated for each target temperature at the time *t* = *T_IR_*. These data are expressed as mean ± SD; for each target temperature sensor output voltage was evaluated three times, as reported in the error bars.

For a given microchannel temperature, the sensor was stimulated with IR radiation, and the voltage (*V_o_*) at *t* = 0.3 ms was evaluated and recorded. Figure 8 displays all the recorded values at the charge amplifier output at the time *t* = 0.3 ms for different microchannel temperatures. Recorded voltages follow a function of *θ*^4^ as predicted by Equation (11). Therefore, by increasing the temperature of the device in the range of 25–65 °C, the total amount of thermal energy proportionally decreases, resulting in a lower pyroelectric charge generation. The analytical and the theoretical results are shown by comparing the pyroelectric peak response versus temperature in Figure 6 and Figure 8. Sensor signal coming out of the charge amplifier is opposite in sign with respect to the analytical response, primarily due to the topology of the preamplifier.

The application of incident IR-shaped radiation on a properly designed pyroelectric transducer and a read-out circuit, combined with a heat transfer model allows measurement of the absolute temperature of an object without the use of adjunctive reference temperature sensors [18,19,20]. The scheme reported in this paper overcomes the problems associated with the evaluation of temperature gradient as previously reported in the literature by using a single dynamic sensor. A comparison among the proposed pyroelectric-based sensor with a selected group of sensors realized using state-of-art material technologies is reported in Table 2. It can be seen from the table that PVDF offers a faster response time even though other materials possess higher maximum working temperatures. However, the temperature range of the pyroelectric sensors is suitable for most microfluidic applications. Investigations of different pyroelectric materials such as PMN-PT crystals have been reported in the literature. These materials possess high-voltage response and potentially represent a promising sensor material. However, they are more expensive compared with PVDF and are typically fragile, which makes them less attractive for commercial applications. Furthermore, PVDF possesses a higher coupling factor than PZT and is also less expensive while demonstrating a high degree of mechanical stability and stable temperature variations. Type T Ultra-Fast Thermocouple can represent a valid counterpart in terms of dimensions and response time for microfluidic applications, although it requires direct contact with the fluid.

Calibration is needed in order to obtain an accurate prediction of the peak output voltage. Close similarities between the analytical and the experimental results prove the consistency of the adopted analysis. Discrepancies can mainly be attributed to the hot chuck setup, the sensitivity of the pyroelectric material to the electromagnetic interference and also to the charge amplifier which produces noise in the output voltage resulting in lowering of the sensitivity. Being a non-linear calibration curve, accuracy is not consistent and error is higher at a lower temperature. Output voltage can be evaluated at a rate defined by the frequency band of the transducer (0.8–49.7 Hz), allowing the evaluation of temperature dynamic inside the microchannel characterized by inherent low thermal inertia at high frequency. Accuracy should be maintained while designing the metallized surfaces in order to fully characterize the device in terms of adsorption and irradiance.

## 4. Conclusions

The research outcomes presented in the paper address the establishment of an innovative way of using pyroelectric thin film for the fabrication of high-performance and cost-effective temperature sensors. Compared to previous works, the actual pyroelectric sensor was developed to evaluate the absolute temperature instead of temperature gradient (with no knowledge about the initial and the final temperatures without the use of additional sensors). In the actual work, the development of an alternate approach involving an IR laser and a thermal model that take into account the heat exchange mechanisms of the active element was presented. Because the pyroelectric transducers do not need to reach thermal equilibrium, there is no latency for the thermalization of the device. This allows the pyroelectric transducers to operate at higher frequencies compared to the traditional thermometers. The period of IR emission, the wavelength, and the power emitted can also be considered to be the key points for optimizing the trade-off between the sensitivity and the temperature frequency acquisition. The performance of the pyroelectric transducer can be remarkably improved and a higher level of integration can be achieved for the realization of the microsystems incorporating low-noise CMOS charge amplifier and filtering stages on a silicon platform. These factors can all play a strategic role in the fabrication of a dedicated lab-on-a-chip for biomedical application. 

## Figures and Tables

**Table 1:** Device parameters of the pyroelectric sensor.

Value		
Active surface [*A*]	10^−6^	[m^2^]
Thermal capacitance [*C_th_*]	32 × 10^−6^	[J·K^−1^]
Absorption coefficient [*α*]	0.7	
Irradiance [*I*]	10^−3^	[W·m^−2^]
Surface emissivity [*δ*]	0.7	
Stefan–Boltzmann constant [*σ*]	5.67 × 10^−8^	[W·m^−2^·K^−4^]
PVDF specific heat [*c_p_*]	1.4 × 10^3^	[J·kg^−1^·K^−1^]
PVDF thermal conductivity [*R_th_*^−1^]	0.2	[W·m^−1^·K^−1^]

**Table 2:** Characteristics of the proposed pyroelectric sensor compared to other sensor technologies.

Technology	Response Time [s]	Dimensions [mm]	Temp. Range [°C]	Reference
**PZT**	5	1.2 × 10^−3^	80–110	[42]
**PZT**	0.00875	1.6 × 10^−3^	31–62	[43]
**PZT**	0.42	0.15	14–93.5	[44]
**LiTaO_3_**	0.01	5.21 × 10^−2^	27–37	[45]
**PMN-30PT**	0.1	8.64 × 10^−3^	32–40.4	[46]
**PMN-25PT**	1	3.76	N/A	[47]
**PVDF**	0.0032	0.25	−40–65	[14,20]
**T-type Thermoc.**	0.005	0.22	−273–150	[48]

## Abbreviations

The following abbreviations are used in this manuscript:

SOC	System-On-a-Chip
PVDF	Polyvinylidene-Fluoride
PZT	Lead Zirconate Titanate
PMMA	Poly(methyl methacrylate)
IR	Infrared
LED	Light Emitting Diode
CMOS	Complementary Metal Oxide Semiconductor
